# Co-Administration of Cholesterol-Lowering Probiotics and Anthraquinone from *Cassia obtusifolia L*. Ameliorate Non-Alcoholic Fatty Liver

**DOI:** 10.1371/journal.pone.0138078

**Published:** 2015-09-16

**Authors:** Lu Mei, Youcai Tang, Ming Li, Pingchang Yang, Zhiqiang Liu, Jieli Yuan, Pengyuan Zheng

**Affiliations:** 1 Department of Gastroenterology, the Second Affiliated Hospital of Zhengzhou University, Zhengzhou, China; 2 Department of Microecology, School of Basic Medical Science, Dalian Medical University, Dalian, China; 3 Medical Microecology and Clinical Nutrition Research Institute of Zhengzhou University, Zhengzhou, China; 4 Longgang Central Hospital, ENT Hospital, Shenzhen ENT Institute, Shenzhen, China; 5 Department of Pathology&Molecular Medicine, McMaster University, Hamilton, Ontario, Canada; Bambino Gesù Children's Hospital, ITALY

## Abstract

Non-alcoholic fatty liver disease (NAFLD) has become a common liver disease in recent decades. No effective treatment is currently available. Probiotics and natural functional food may be promising therapeutic approaches to this disease. The present study aims to investigate the efficiency of the anthraquinone from *Cassia obtusifolia L*. (AC) together with cholesterol-lowering probiotics (P) to improve high-fat diet (HFD)-induced NAFLD in rat models and elucidate the underlying mechanism. Cholesterol-lowering probiotics were screened out by MRS-cholesterol broth with ammonium ferric sulfate method. Male Sprague–Dawley rats were fed with HFD and subsequently administered with AC and/or P. Lipid metabolism parameters and fat synthesis related genes in rat liver, as well as the diversity of gut microbiota were evaluated. The results demonstrated that, compared with the NAFLD rat, the serum lipid levels of treated rats were reduced effectively. Besides, cholesterol 7α-hydroxylase (CYP7A1), low density lipoprotein receptor (LDL-R) and farnesoid X receptor (FXR) were up-regulated while the expression of 3-hydroxy-3-methyl glutaryl coenzyme A reductase (HMGCR) was reduced. The expression of peroxisome proliferator activated receptor (PPAR)-α protein was significantly increased while the expression of PPAR-γ and sterol regulatory element binding protein-1c (SREBP-1c) was down-regulated. In addition, compared with HFD group, in AC, P and AC+P group, the expression of intestinal tight-junction protein occludin and zonula occluden-1 (ZO-1) were up-regulated. Furthermore, altered gut microbiota diversity after the treatment of probiotics and AC were analysed. The combination of cholesterol-lowering probiotics and AC possesses a therapeutic effect on NAFLD in rats by up-regulating CYP7A1, LDL-R, FXR mRNA and PPAR-α protein produced in the process of fat metabolism while down-regulating the expression of HMGCR, PPAR-γ and SREBP-1c, and through normalizing the intestinal dysbiosis and improving the intestinal mucosal barrier function.

## Introduction

With the prevalence of obesity, hyperlipidemia, type II diabetes, and metabolic syndrome, the morbidity of nonalcoholic fatty liver disease (NAFLD) is constantly rising worldwide [1]. Recent studies indicate that about 50% of NAFLD patients may develop into non-alcoholic steatohepatitis (NASH) and 40% may progress to liver fibrosis after 4–13 years [2]. The pathophysiological process of NAFLD is extremely complicated and the pathogenesis is unclear. No effective cures have been found currently with the prognosis of NASH being pessimistic. Therefore, there is an urgent need to develop effective remedies for patients suffering NAFLD.

NAFLD patients are usually accompanied with obesity and insulin resistance, but not all obese people develop into NAFLD, the intestinal factors may play a key role in the pathogenesis of NAFLD [3]. Studies have shown that the lipopolysaccharide (LPS) of gram-negative bacteria in the intestine can be transported to the liver through the portal vein. Under condition of dysbiosis, a large amount of Gram-negative bacteria may over proliferate and produce endotoxins, results in metabolic disorders, obesity, diabetes, NALD and NASH [4]. Changes of intestinal microbiota in the acute liver injured mice resulted in increased intestinal permeability and bacterial translocation [5]. In addition, the reaction of the mouse to high-fat diet (HFD) can be determined by the different composition of the intestinal microbiota and levels of circulating endotoxin level, which are significantly higher in NAFLD patients with excessive growth of intestinal bacteria and enhanced intestinal permeability [6, 7]. Since the intestinal microbiota is closely related to obesity, insulin resistance, NAFLD and NASH, it is possible to prevent the occurrence of NAFLD by adjusting the intestinal microbial structure.

Probiotics and Chinese herb medicine may be promising approaches to affect intestinal microbiota. There have been studies on the capacity of probiotics to attenuate the unbalanced homeostasis and cholesterol levels in the body [8, 9]. Many probiotics were made into yogurt using as food supplements for patients with hyperlipidemia and NAFLD [10]. On the other hand, adding natural functional food additives with the characteristic of inhibiting fat accumulation can result in satisfactory effect on the improvement of obesity, insulin resistance and other metabolic diseases [11], which provides a new direction for the treatment of NAFLD. *Cassia obtusifolia L*. belongs to a leguminous annual herb in tropical countries in Asia, its main active ingredients are anthraquinone compounds, including obtusin, emodin and aloe-emodin [12]. Its herbal ingredients are popular as a kind of functional beverage with the effects of reducing serum levels of fat and cholesterol, anti-oxidation, anti-fungal, and neuroprotection [13, 14]. *Cassia obtusifolia L*. can also protect the liver function in rats with liver injury, alleviate obesity, insulin resistance and NAFLD by up-regulating the AMP-dependent protein kinase [15, 16].

Although both probiotics and anthraquinone of *Cassia obtusifolia L*. (AC) have beneficial effects for meliorating metabolic diseases such as obesity, insulin resistance and NAFLD, it is still unclear whether application of them together as a symbiotic formulation could enhance the NAFLD-preventing effect. We hypothesized that by combining the probiotics and AC, the preventive or improving effects on NAFLD can be enlarged, and this may associated with the improvement of the intestinal microbial structure and metabolism of energy by the liver. To test the hypothesis, the screening and characterization of cholesterol-lowering probiotics were conducted. Then, by treating the selected probiotics together with AC, the effects of the symbiotic formulation on HFD-induced NAFLD rats were tested.

## Materials and Methods

### Material

AC was provided by Chongqing Academy of Chinese Materia Medica[17]. The Ammonium iron (III) sulfate dodecahydrate and Cholesterol were purchased from Sigma (St. Louis, MO, USA). The growth medium De Man Rogosa and Sharpe (MRS) broth were purchased from Difco (USA).

### Screening and characterization of cholesterol-lowering probiotics

The lactic bacteria (LAB) strains isolated from fermented food or healthy human intestine were screened by MRS-cholesterol broth with the Ammonium ferric sulfate method [18]. The properties of selected candidate strains, including tolerance to acid, bile, pepsin and trypsin, cell adhesion ability were evaluated [19]. They were identified by 16S rDNA sequencing and the sequences were submitted to NCBI under the GenBank Accession No. KP967559-KP967561.

### Animals experiments

30 male Sprague–Dawley rats (weighted of 120-140g) were obtained from the SPF animal center of Dalian Medical University. After acclimatization for 1 week on a standard diet, the rats were divided randomly into 5 experimental groups (6/group). Group 1 received a normal diet (ND) (10% of calories derived from fat; D12450B). Group 2 received a HFD (45% of calories derived from fat diet; D12451) to establish NAFLD models (All feeds purchased from Research Diets, New Brunswick, NJ). Group 3 received HFD containing AC (0.2g/kg per day) [16]. Group 4 received HFD containing cholesterol-lowering probiotics (P) (2×10^10^ CFU/ml in 0.9% NaCl, 1 ml per day). Group 5 received HFD containing AC and P. Rats were euthanized on day 150 after the diet regimens were completed, and subjected to morphological, biochemical, and molecular biological analyses. The study protocol was approved by the Animal Care Committee of the Dalian Medical University, China (SCXK-2008-0002).

### Histological examination

Specimens were taken from the liver and intestine, and were fixed in 4% formaldehyde for 24 h. The tissue was embedded in paraffin and cut into 5 mm sections used for H&E staining. A piece of the liver was snap frozen in liquid nitrogen; the cryosections were prepared for Oil Red-O staining.

### Biochemical analyses

The overnight-fasted rats were sacrificed by decapitation; the trunked blood was collected in heparinized tubes, and centrifuged at 2,000×g at 4°C for 20 min. The serum was collected and analyzed for total cholesterol (TC), total triacylglycerol (TG), high-density lipoprotein (HDL), low-density lipoprotein (LDL), free fatty acid (FFA) and insulin (INS) by commercial reagent kits (Jiancheng, China) according to the manufacturers’ instructions. Hepatic tumor necrosis factor-α (TNF-α) level was measured by ELISA using the Quantikine Rat ELISA kit (R&D Systems, Minneapolis, USA). Peripheral blood glucose concentration were determined using an automated glycemia reader (Accu-Chek Active Blood Glucose Meter, ROCHE, Germany). Homeostasis model assessment of insulin resistance (HOMA-IR) was calculated using the following formula: [immuno-reactive insulin (mIU/L) ×fasting blood sugar (mmol/L) ÷22.5] [20]. For assay of endotoxin, the blood samples were collected from the portal vein, and endotoxin were determined using the Limulus Amebocyte Lysate kit (LAL factory in Xiamen, China) according to the manufacturer’s instructions.

### Gene expression analysis by quantitative real-time PCR (qPCR)

Liver and intestine samples of rats were collected for mRNA quantification. Total RNA was extracted using a RNAiso Plus kit (Takara, JAPAN) and was reverse transcribed into cDNA using a PrimeScript™ RT Master Mix (Takara, JAPAN). The expression levels of CYP7A1, LDL-R, HMGCR in liver and FXR in the intestine were analyzed by qPCR by SYBR® Premix Ex Taq™ (Takara, JAPAN), the primers and PCR conditions are summarized in **S1 Table**.

### Western blotting

The total protein was extracted from liver and intestine. Equal amounts of proteins were fractioned with sodium dodecylsulfate polyacrylamide gel electrophoresis (SDS-PAGE) followed by electrophoretic transfer of proteins onto nitrocellulose membranes. The blots were probed with antibodies against PPAR-α, PPAR-γ, SREBP-1c, ZO-1, Occludin (Abcam, Cambridge, UK), and followed by incubation with secondary antibodies conjugated with horseradish peroxidase (HRP; ThermoFisher, USA). The immune complexes were detected with a WesternBright™ ECL Western Blotting HRP Substrate kit and analyzed with image lab software (Bio-Rad, USA).

### Sequencing of the V4 region of 16S rDNA gene

Microbial genomic DNA was extracted from fecal samples using E.Z.N.A®. Mag-Bind® Stool DNA Kit (OMEGA, USA). The V4 hypervariable region of 16S rRNA gene were amplified using the primers: 5’-ACTCCTACGGGAGGC-AGCAG-3’ and: 5’-AYTGGGYDTAAAGNG-3’). PCR product was excised from a 1.5% agarose gel and purified by the QIAquick Gel Extraction Kit (QIAGEN, Germany) and was sequenced using pair-end method by Illumina Miseq with a 6 cycle index read. Each sample’s trimmed sequence was compared to sequences were assigned to different taxonomic levels (from phylum to species) using the Greengene database [21]. Using QIIME, sequences were further clustered in at 97% of identity in operational taxonomic unit (OTU) using uclust [22]. OTU were assigned to the closest taxonomic neighbours and relative bacterial species using blast and up-to-date 16S rRNA gene RDP database [23, 24]. Estimates of phylotype richness were calculated according to the bias-corrected Chao1 estimator. Principal component analyses (PCA) with every group at different time points as instrumental variables (intraclass PCA) were computed and statistically assessed.

### Statistical analysis

All data were evaluated as the mean ± standard deviation (SD). Statistical analysis of the quantitative multiple group comparisons was performed using the one-way analysis of variance (ANOVA) followed by Duncanʼs test; whereas pairwise comparisons were performed using the t test by SPSS 17.0 system (SAS Institute Inc, USA) and GraphPad Prism 5 (Graph Pad Software, La Jolla, CA, USA). Results were considered to be statistically significant with p<0.05.

## Results

### Cholesterol-lowering strains DM9054 and 86066 were identified

From the collection of lactic acid bacteria which were originally isolated from the traditional Chinese fermented foods or feces of healthy humans, 7 strains with cholesterol-lowering efficiency over 55% were screened out. They were co-cultured with AC to test the cholesterol-lowering activity. As shown in Table 1, the cholesterol-lowering efficiency of DM9054, DM9073 and 86066 was improved significantly. The acid tolerance, bile tolerance (**S2 Table**) and adhesion ability to Caco-2 cells (**S1 Fig**) of DM9054, DM9073 and 86066 were further evaluated. Results showed that DM9054, 84034 and 86066 survived at pH 2, and strains 8503 and 84031 were unable to survive in 0.3% bile salts, while DM9007, DM9054 and 86066 had the best cell adhesion abilities. Therefore, DM9054 (*Lactobacillus Rhamnosus GG*, *LGG*) and 86066 (*Lactobacillus plantarum WCFS1*, *LP*) were selected as the candidate strains for further research.

**Table 1:** The abilities of candidate strains to reduce cholesterol.

Strains	Strains in different media of cholesterol degradation rate (48h)	
	MRS-CHOL(%)	MRS-CHOL+TAC(%)
**DM9007**	55.87±0.26 ^a^	58.62±0.45 ^a^
**DM9054**	55.48±0.40^b^	65.21±0.27 ^a^
**DM9073**	56.04±0.35^b^	62.21±0.33 ^a^
**8503**	56.52±0.27 ^a^	61.97±0.31 ^a^
**84031**	55.11±0.23^a^	56.97±0.41 ^a^
**84034**	59.91±0.29 ^a^	56.53±0.25 ^a^
**86066**	57.71±0.34^b^	68.69±0.47 ^a^

MRS-CHOL, MRS broth at a final concentration of cholesterol was 0.1mg/ml. Results are expressed as mean ± SD (n = 7). Means within a row with different superscript letter significantly different (P <0.05)

### Co-administration of DM9054, 86066 and AC reduced blood lipid levels and improved IR of rats

**Fig 1:**
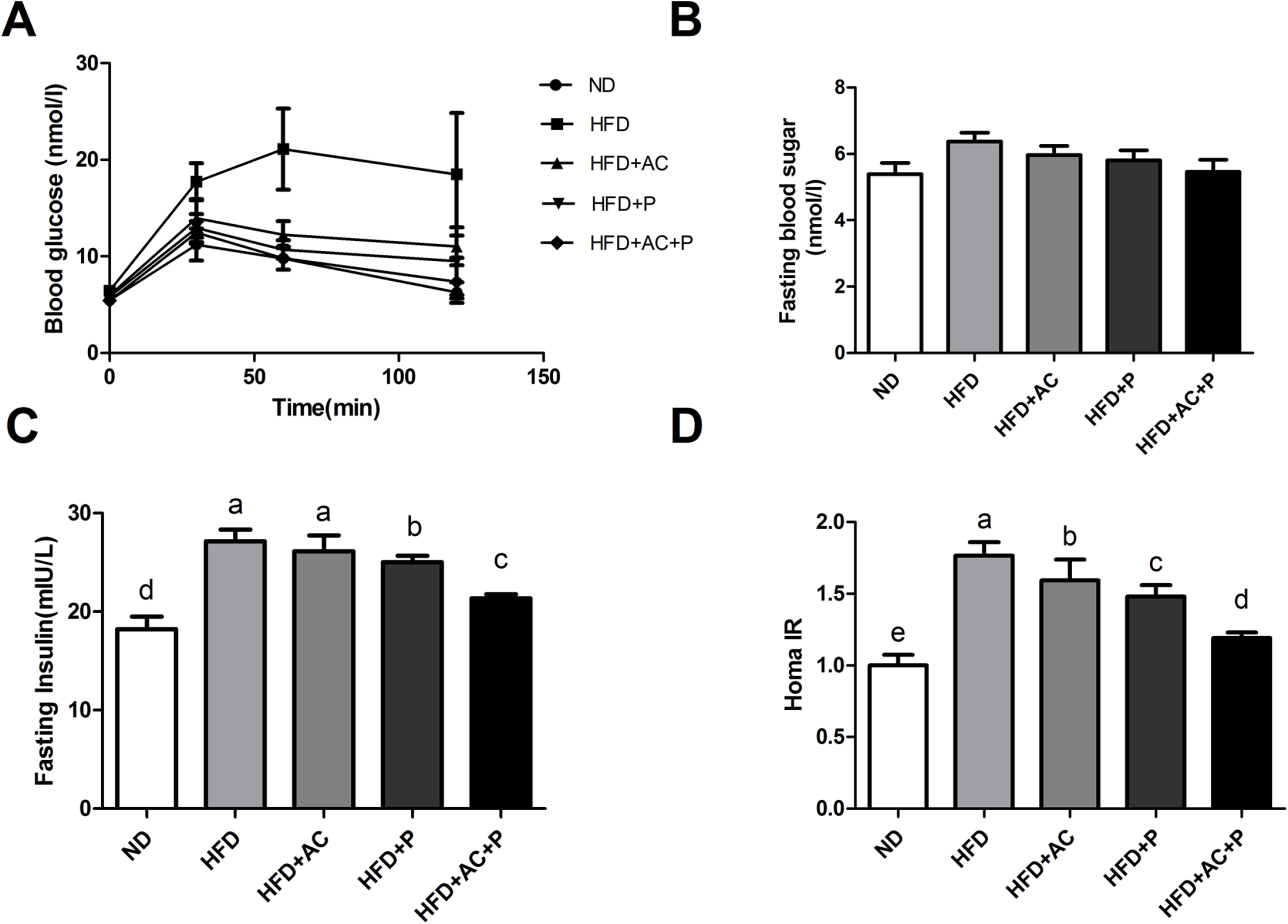
Probiotics combined with AC ameliorated blood glucose levels and IR. (A) Intraperitoneal glucose tolerance test. (B) Fasting blood glucose levels. (C) Fasting plasma insulin levels, and (D) HOMA-IR were assessed. The data are shown as mean±SD, n = 6. Means within a row with different superscript letters are significantly different (P<0.05).

The effects of co-administration of DM9054 and 86066 with AC on blood lipids of rats fed with HFD were given in Table 2. Compared with the ND group, the serum levels of TC, TG, LDL and FFA of HFD Group increased significantly, while serum HDL decreased greatly (P<0.05). Compared with the Group HFD, the serum TC, TG, LDL and FFA of Group HFD+AC and Group HFD+P decreased, while serum HDL increased (P<0.05), and the changes of these parameters in Group HFD+AC+P were more obvious. After administration of probiotics DM9054, 86066 and/or AC, the abnormal intraperitoneal glucose tolerance of HFD rats was significantly improved (**Fig 1A**). The five groups had no significant difference regarding the levels of fasting blood glucose (P>0.05) (**Fig 1B**). The IR of Group HFD+AC, Group HFD+P and Group HFD+AC+P was effectively improved, and among the groups, the IR-improving effect of AC+P was the most remarkable (P<0.05, **Fig 1C and 1D**).

**Table 2:** Serum TC, TG, HDL, LDL, and FFA concentrations in SD rats fed the experimental diets.

Lipid profile	ND	HFD	HFD+AC	HFD+P	HFD+AC+P
**TC(mg/dl)**	127.95±4.52^d^	223.12±5.61^a^	179.88±4.29^b^	160.96±6.87^bc^	150.62±5.63^cd^
**TG(mg/dl)**	75.15±4.28^d^	136.86±5.64^a^	111.93±4.22^b^	120.29±5.14^b^	102.45±4.39^c^
**LDL(mg/dl)**	57.33±3.67^d^	156.65±5.96^a^	121.73±3.89^b^	112.06±3.91^b^	89.93±3.57^c^
**HDL(mg/dl)**	76.07±3.05^a^	49.01±3.12^d^	53.96±3.06^c^	59.97±4.79^b^	62.41±2.26^b^
**FFA(mg/dl)**	29.42±3.58^c^	60.94±4.28^a^	46.85±3.91^b^	42.41±3.14^b^	37.37±3.87^bc^
**LDL/HDL ratio**	0.75±0.34^e^	3.20±0.32^a^	2.27±0.15^b^	1.88±0.29^c^	1.44±0.11^d^
**TC/HDL ratio**	1.72±0.21^d^	4.49±0.41^a^	3.31±0.49^b^	2.72±0.19^c^	2.39±0.23^c^

TC, total cholesterol; TG, total triacylglycerol; LDL, low-density lipoprotein; HDL, high-density lipoprotein; FFA, free fatty acid.

Results are expressed as mean±SD, n = 6. Means within a row with different superscript letters are significantly different (P<0.05).

### Co-administration of DM9054, 86066 and AC reduced hepatic steatosis in rats

**Fig 2:**
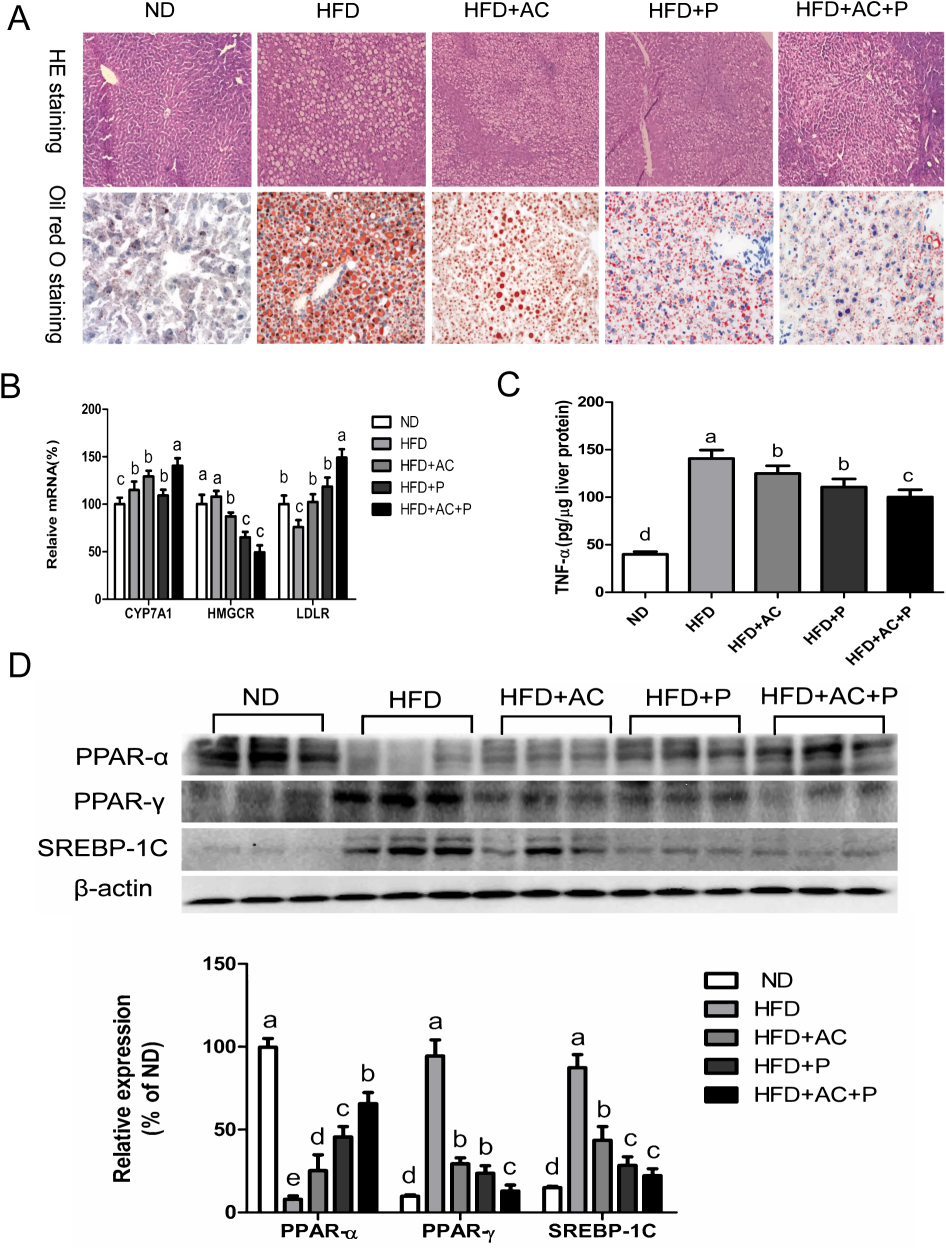
Probiotics and AC reduced hepatic lipid deposition. (A) HE and oil red O staining of liver tissues. Data are representative of 6 individual liver sections. Original magnification, ×100. (B) Hepatic mRNA expression levels of HMGCR, LDL-R, and CYP7A genes. Results are expressed as mean±SD (n = 6). (C) Hepatic TNF-α level was analyzed by ELISA. Data are expressed as mean±SD (n = 6). (D) PPAR-α, PPAR-γ, SREBP-1C expression were detected by western blotting. The ß- actin was used as a loading control. Data are expressed as mean±SD (n = 3). All mean values within treatment groups with different lowercase letters are significantly different (P < 0.05).

By HE staining and oil red O, it was found that the liver of Group HFD showed moderate hepatic steatosis, a slight disorder of globular structure and the cytoplasm was full of small lipid droplets or foamy lipid droplet vacuoles, the scattered chronic inflammatory cells were seen in the infiltration. While the fatty degeneration and fat deposition of the other three intervention groups were improved and the effect of AC+P was the most significant (**Fig 2A**). The mRNA expression of genes involved in the regulation of liver cholesterol metabolism, including genes encoding HMGCR, LDL-R, CYP7A1, and β-actin was detected by qPCR. The expression levels of CYP7A1 and LDL-R in Group HFD decreased significantly, while in the three intervention groups, the levels of CYP7A1 and LDL-R were higher, opposite to the lower expression level of the HMG-CR mRNA. Among these three groups, the intervention of AC+P was the most effective (P<0.05, **Fig 2B**). TNF-α play an important role in hepatic steatosis, HFD-induced increase in hepatic TNF-α production was significantly suppressed by cholesterol-lowering probiotics combined with AC treatment (P<0.05, **Fig 2C**). The expression of PPAR and SREBP-1C in liver of rats was detected by western blot. Result showed that PPAR-α expression in liver of rats fed with HFD increased drastically (P<0.05), while the levels of PPAR-γ and SREBP-1C reduced significantly (P<0.05). Among the three intervention groups, increased expression of PPAR-α, decreased expression of PPAR-γ and SREBP-1C were detected. Remarkable differences were detected between the HFD+AC+P group and other groups (all P<0.05, **Fig 2D**).

### Co-administration of DM9054, 86066 and AC improved the intestinal mucosal barrier function, reduced the level of endotoxin and regulated the expression of FXR

**Fig 3:**
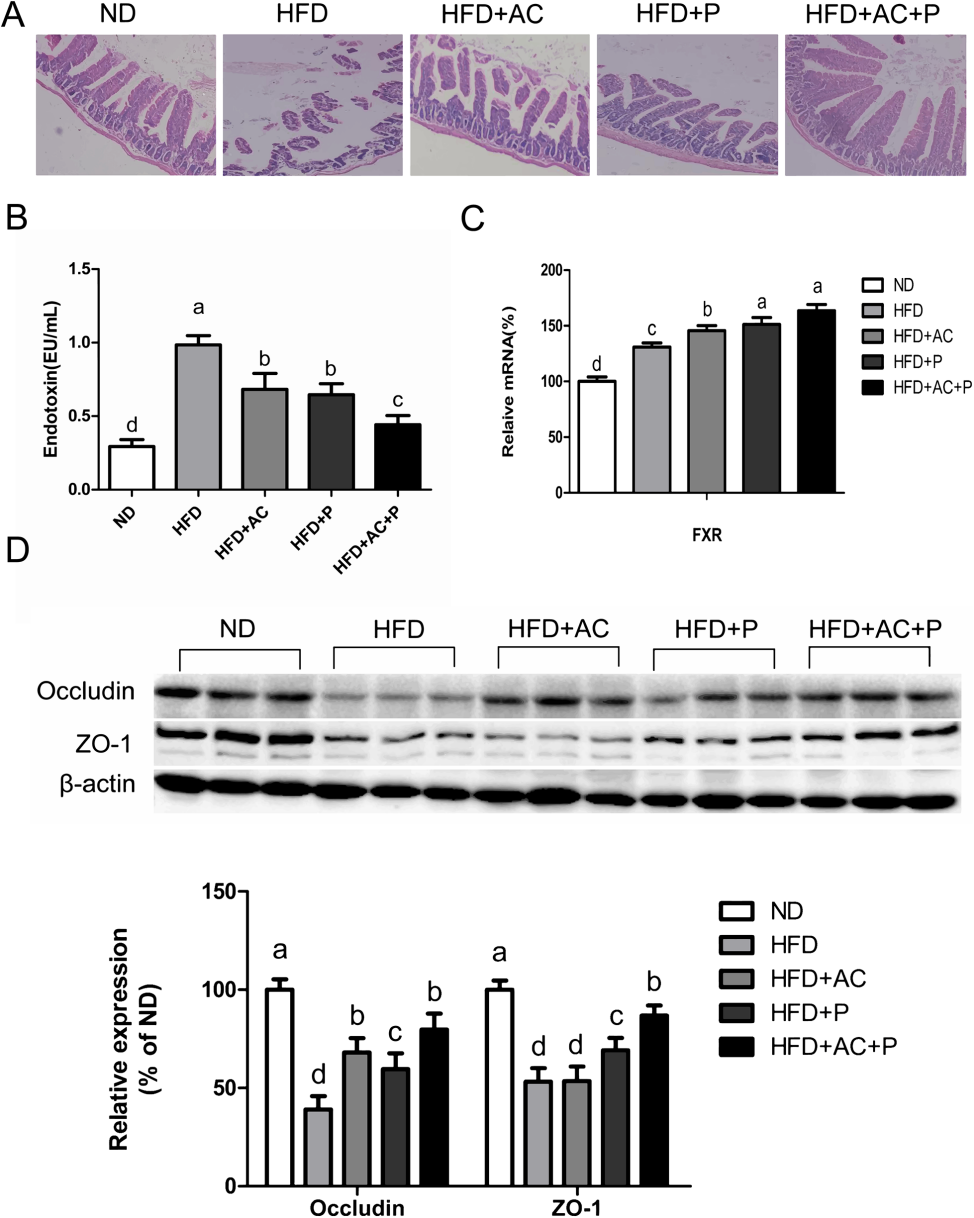
Probiotics and AC reduced endotoxin levels in rats and promoted ZO-1, occludin expression, and FXR mRNA expression. (A) Pathological morphology observed in rat ileum, Original magnification, ×100 (B) Serum levels of endotoxin. Data are expressed as mean±SD(n = 6). (C) Intestinal mRNA expression levels of the FXR genes. Results are expressed as mean±SD (n = 6) (D) Expression of ZO-1 and occludin were analysed by western bloting. The ß-actin expression was used as a loading control. Data are expressed as mean±SD (n = 3). All mean values within treatment groups with different lowercase letters are significantly different (P < 0.05).

The intestinal mucosal barrier function, intestinal endotoxemia and FXR expression of ileum play crucial roles in NAFLD progression. HE staining results showed that the intestinal mucosal villi of rats in Group ND were slim, neatly arranged and the surface structure was integral with no congestion, edema and other changes. On the contrast, the intestinal mucosal villi of rats in HFD Group appeared rupture, missing, and the epithelial cell necrosis were founded. Through the intervention of probiotics or AC, the arrangement of the villi of the three intervention groups had recovered, in which the effect of AC+P was the most obvious (**Fig 3A**). The plasma level of endotoxin in HFD rats was obviously higher than Group ND, while through the intervention of probiotics and AC, it decreased significantly (**Fig 3B**). When detecting the expression of ileum FXR mRNA, significant higher level of FXR mRNA expression in Group HFD was observed, which was about 1.3 times of Group ND, while the FXR expression level in the intervention group increased even higher, which was about 1.5–1.6 times of normal level (**Fig 3C**).

Intestinal permeability is the main factor of the intestinal mucosal barrier function which is mainly regulated by tight-junction (TJ) proteins, including ZO-1 and occludin. Western-blotting results showed that compared with Group ND, the expression of ZO-1 and occludin of Group HFD decreased significantly, and increased in the three intervention groups, in which the effect of AC+P was the most obvious (**Fig 3D**).

### Co-administration of DM9054, 86066 and AC affected intestinal microbial structure and diversity

**Fig 4:**
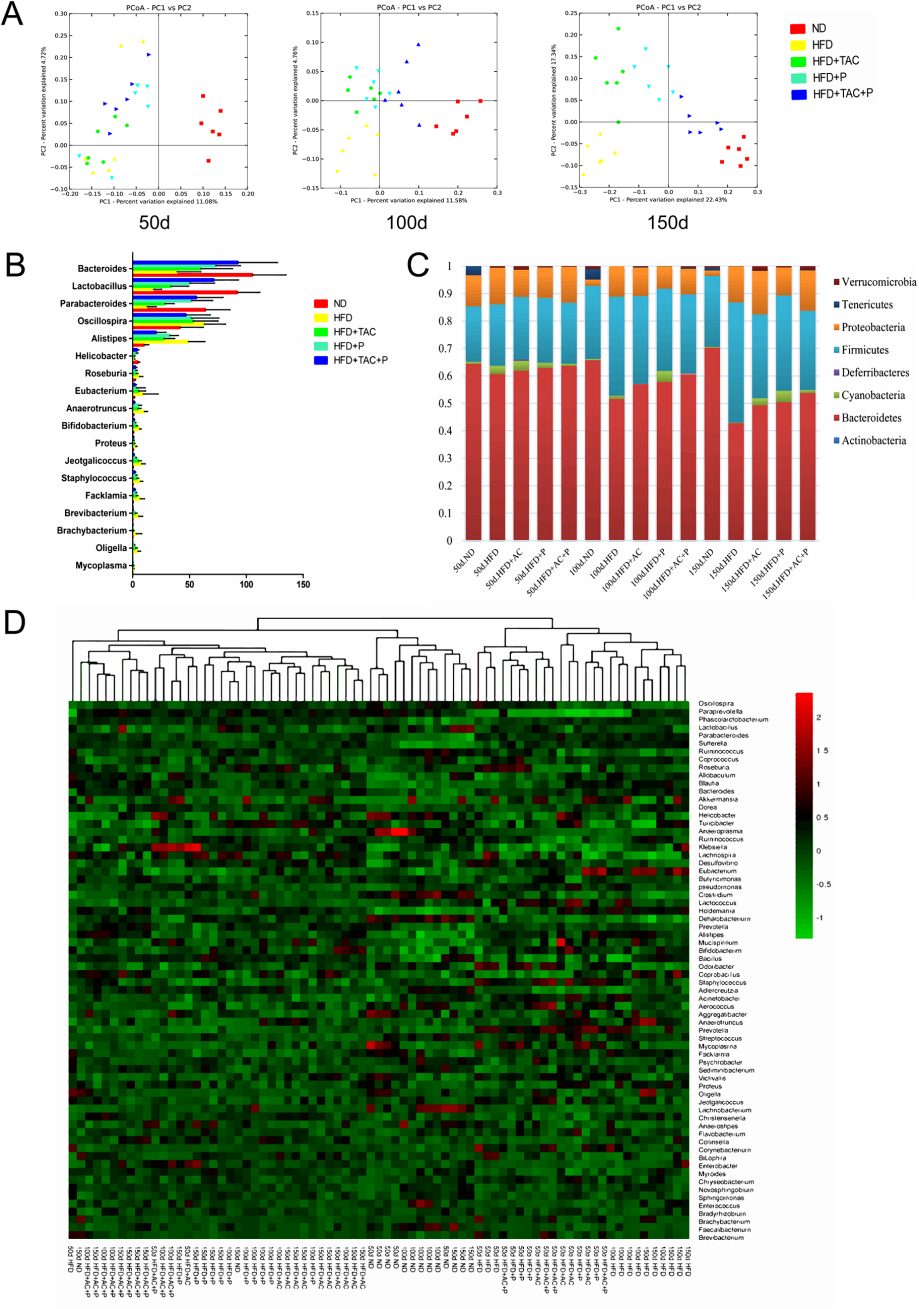
Probiotics and AC regulates the intestinal microbial composition and diversity. (A) PCA in the 50^th^ day, the 100^th^ day and the 150^th^ day were performed based on the genera abundance of the microbial genomes. (B) Main genus composition was a percentage of total assigned sequences. (C) Bacterial phyla distribution as a percentage of total sequences. (D) Heatmap represents a sample of a color, abundance of a genus is a longitudinal sample clustering situation, reflecting on the case of multiple samples at the level of community composition similarity. All presented results are statistically significant (p<0.05) as assessed by the Wilcoxon test.

Caecal samples from receiver rats on day 50, day 100 and day 150 were analyzed by pyrosequencing. A total of 7 583 846 sequences was obtained and after trimming, 5,740,971 sequences were further analyzed. To evaluate similarity among 50 day, 100 day, 150 day samples, interclass PCA was performed based on their microbial composition, and the cluster analysis was performed (**Fig 4A**). Results showed that Group HFD+AC+P was closer to the normal group. The levels of the commensal microbes of rats in the five groups were detected, it turned out that the *Firmicutes* of Group HFD increased obviously while the *Bacteroidetes* decreased compared with the normal group. The intervention of P+AC attenuated the dysbiosis of rat intestine, as seen the population of *Bacteroides* increased and the population of *Firmicutes* decreased (**Fig 4C**). When analyzing the intestinal microbial taxa of rats, we found that the *Bacteroides*, *Lactobacillus* and *Parabacteroides* of Group HFD decreased greatly compared with Group ND, while *Oscillospira* increased obviously (all P<0.05), and the intervention of probiotics or AC effectively increased *Bacteroides*, *Lactobacillus* and *Parabacteroides* and decreased *Oscillospira*. Among the intervention groups, the effect of AC+P was the most obvious (**Fig 4B**). The heat map analysis further confirmed our results (**Fig 4D**).

## Discussion

The current NAFLD therapies include lifestyle modifications, physical activity and medical intervention in general [25, 26]. However, the long-term lifestyle modification and physical activity is hardly to be carried out, while most of the medicines have adverse effects which limit using. Thus, it is necessary to explore novel remedies with less side effects and higher therapeutic effect. A probiotic and functional food dietary intervention could be a promising and cost effective approach in the management of NAFLD. Many probiotics were applied to regulate lipid metabolism, but have rarely been tested for their cholesterol-lowering potential. Our data show that by combining the cholesterol-lowering probiotics DM9054, 86066 and AC, the development of HFD-induced NAFLD in rat was effectively prevented, and the effect of co-administration was more significant compared with either probiotics or AC. However, metabonomics and proteomics study of cholesterol-lowering probiotics alone and in combination with AC can prevent NAFLD remains unclear, need further studies.

Hypercholesterolaemia, hypertriglyceridaemia, low HDL levels, and high LDL levels are the most common impairments in lipid homeostasis in patients with hepatic steatosis [27]. Although it was not replicate the full spectrum of the disease in humans, high-fat diet induced animal model of NAFLD has been widely used to identify the pathogenesis and evaluate new treatments for NAFLD. In our model, HFD-fed rats developed hepatic steatosis, visceral obesity, hyperlipidaemia, and increased FFA, which mimics almost all of the clinical aspects of human NAFLD [28]. We observed that cholesterol-lowering probiotics alone and in combination with AC reduced the levels of TG, TC, LDL-C, and significantly suppressed FFA in the plasma, whereas the plasma HDL level was obviously elevated in HFD-fed rats. Given that the LDL/HDL ratio is positively correlated with the risk of coronary heart disease, even when TC concentrations are elevated [29], and that the TC/HDL ratio is a sensitive predictor of atherosclerosis [30], the low ratios in HFD-fed rats treated with probiotics alone and in combination with AC suggest that probiotics alone and in combination with AC has anti-atherogenic potential. Meanwhile, histological evidence suggests that probiotics alone and in combination with AC significantly prevented hepatic lipid accumulation in HFD-fed rats. This beneficial effect may be positively correlated with reductions in TG, TC, FFA, and LDL, and an elevation in HDL synthesis. Administration of probiotics alone and in combination with AC significantly suppressed the increase of IR in dietary obese rats after 150 days, suggests that the HFD-induced hepatic steatosis maybe ameliorated via down-regulation of lipid accumulation in the plasma and liver, and improvement of IR and glucose tolerance.

The cholesterol levels in the blood are regulated at different levels, including absorption, synthesis, and excretion. However, the LDL receptor–mediated endocytosis controls plasma cholesterol levels by hepatic absorption, and the LDL receptor is regulated by a transcriptional control mechanism [31]. A regulatory enzyme, HMG-CoA reductase, in the cholesterol synthesis pathway catalyzes the synthesis of mevalonate from HMG-CoA and is regulated at the post-transcriptional level [32]. Fecal bile excretion is the only direct path for decreasing the level of cholesterol, and it is regulated by CYP7A1 and FXR [33, 34]. The expression levels of HMGCR and LDL-R represent the cholesterol inputs to the liver, although the bile acid synthesis (CYP7A1 and FXR) is an indication of a decrease in hepatic cholesterol. HFD decreases the input of cholesterol to the liver, especially that from cholesterol synthesis. The reason may be that the HFD causes too high a cholesterol level in the rat body, which is a negative feedback on the HMGCR occurrence to prevent more cholesterol synthesis [35]. The present study demonstrated that supplementation with probiotics and/or AC inhibited the HMGCR expression compared with the HFD group. Cholesterol absorption through LDL-R was found to be affected by dietary cholesterol supplementation, however, the LDL-R mRNA expression was up-regulated in the group supplemented with probiotics and/or AC. The reaction by CYP7A1 is a rate-limiting step in bile acid synthesis from cholesterol, and its expression and activity can be increased by endogenous and dietary cholesterols. HFD supplementation increased bile acid synthesis and supplementation with probiotics and/or AC also increased the CYP7A mRNA expression. However, FXR could sense the bile acid, which is secreted from the bile duct and flowed into the intestinal tract, and then this information is feedback to the liver to slow down both CYP7A1 expression and excessive generation of bile acid [33, 36]. We observed that, compared with the ND group and the HFD group, there was up-regulation of FXR expression in probiotics and/or AC groups, which suggests more stimulation in the bile acid environment in the intestinal tract to keep dynamic equilibrium at a higher level with CYP7A1 expression; in this way, it can prevent excessive bile acid generation. Therefore, the mechanism by which the administration with probiotics alone or in combination with AC improves NAFLD may be promoting via the excretion and absorption of cholesterol in the liver to reduce cholesterol synthesis.

PPAR controls the expression of many target genes that are involved in the lipid metabolism and among these genes, PPAR-γ is mainly related to the fat formation while PPAR-α mainly controls fat absorption and β oxidation in peroxisome [37]. The present study demonstrated that there were significantly lower PPAR-α expression levels in the rats’ livers of the HFD group than those in the normal diet group while PPAR-γ expression levels were significantly higher than that in the ND group. It is also observed that for all rats adopting HFD, there was up-regulation of PPAR-α levels, but down-regulation of PPAR-γ levels with a significant difference in probiotics and/or AC treated groups. Wang, *et al*. [38] also found that there was an increase of FFA content and hepatic lipid in the serum and liver of the PPAR-α-deficient rats. Besides, SREBP-1C is closely related to fatty acid metabolism and glycometabolism, and is the major transcriptional regulation factor for the fat synthesis genes [39]. Under the normal condition, there is a low SREBP-1C expression level in the rat liver but after the adoption of HFD, there may be a significant rise in the expression of SREBP-1C, which has a close relationship with NAFLD [40]. After the application of probiotics alone or in combination with AC, it is found that there was a significant decline in the SREBP-1C levels, which greatly improved in the fatty liver. It may be that the overexpression of FXR inhibits SREBP-1C in the triglyceride generation [41].

Different dietary composition can modulate the divergent composition of gut microbiota[42]. The changes in the overall composition of gut microbiota induced by HFD versus normal chow may act as an important mediator in the etiology of NAFLD and related metabolic diseases via disrupting the host lipid metabolism regulation and inducing low-grade inflammation[43]. In the present study, we also observed the impact of AC on the composition of the intestinal microbiota, it seems to play a role in the prevention of NAFLD. This conclusion can be drawn from the fact that AC induced an increase of the total amount of the intestinal microbiota and, especially, a shift towards the beneficial bacteria phyla *Firmicutes* and *Bacteroidetes*. Previously, there had not been such a report on the AC impact on the intestinal microbiota. The herein described effects of AC may thus be indirectly due to an attenuation of the altered barrier function caused by HFD. Indeed, we found that the expressions of major tight junction proteins, occludin, are enhanced if AC is administered to HFD-receiving rats. In addition, AC also inhibits the inflammatory activity of TNF-α so as to improve NAFLD. TNF-α and IL-6, important cytokines related with live inflammation, were considered as the factors that contribute to steatohepatitis. Especially, TNF-α mediates the early stage of fatty liver disease as well as the transition to a more advanced stage of liver disease, and stimulates the release of cytokines such as IL-4 and IL-6[44]. Research indicated that the serum or plasma TNF-α and IL-6 levels was higher in NASH compared healthy subjects and it was shown to correlate with liver fibrosis in advanced NAFLD [45, 46]. Although IL-6 was not detected in our study, the TNF-α results still suggested that the beneficial effects of AC on the intestinal barrier function, possibly result in the here shown decreased translocation of LPS from the gut to the liver and thus a decreased liver inflammation and steatosis. This may be a new mechanism for AC improve NAFLD, suggesting AC metabolic process can serve as a regulator of the intestinal microbiota, which in turn reduces the release of inflammatory cytokines and decreases the translocation of LPS to prevent NAFLD. As for probiotics, the above results have been reported [47, 48], and as shown earlier, this improvement of NAFLD has been strengthened at the cholesterol-lowering probiotics combination with AC.

In conclusion, the present data show that the cholesterol-lowering probiotics alone and in combination with AC protect against HFD-induced NAFLD. The underlying mechanisms involve not only keeping a homeostasis of the intestinal microbiota, modulation of intestinal barrier function and reducing intestinal endotoxemia, but also decreasing the release of inflammatory cytokines, regulation of lipid metabolism and improving IR through increasing the expression levels of both CYP7A1 and LDL-R in the liver, decreasing the expression levels of HMG-CR, up regulating PPAR-α levels while down regulating PPAR-γ and SREBP-1C levels. Therefore, an optimized blend of the cholesterol-lowering probiotics and AC could be exploited as a potential biotherapeutic remedy to decrease cholesterol levels and lower the risk of NAFLD, although the field is open for further studies.

## Supporting Information

S1 ARRIVE Guidelines Checklist The ARRIVE Guidelines Checklist Animal Research: Reporting In Vivo Experiments. (PDF)

S1 Fig Adhesion abilities of the probiotics to Caco-2 cells. The adhesion scores indicate values of bacteria cells adhered to one Caco-2 cell. All values are means±SD, n = 7.
(TIF)

S1 Table List of primer sequence for reverse transcriptase polymerase chain reaction and the amplicons obtained for different genes. (DOCX)

S2 Table The tolerance of candidate strains to biological barriers (Log CFU/mL). ^a^Each value represents mean±SD, n = 7. ^b^- No growth.
(DOCX)

S3 Table Effect of *Cassia obtusifolia L*. on fecal properties of SD rats. Data are mean±SD values (n = 6). Means within a row with different superscript letters are significantly different (P<0.05).
(DOCX)
