# Supplementary figures and images for: Co-Administration of Cholesterol-Lowering Probiotics and Anthraquinone from Cassia obtusifolia L. Ameliorate Non-Alcoholic Fatty Liver
Source: PLoS One. 2015 Sep 16;10(9):e0138078. doi: 10.1371/journal.pone.0138078 (PMC4573521; doi:10.1371/journal.pone.0138078)

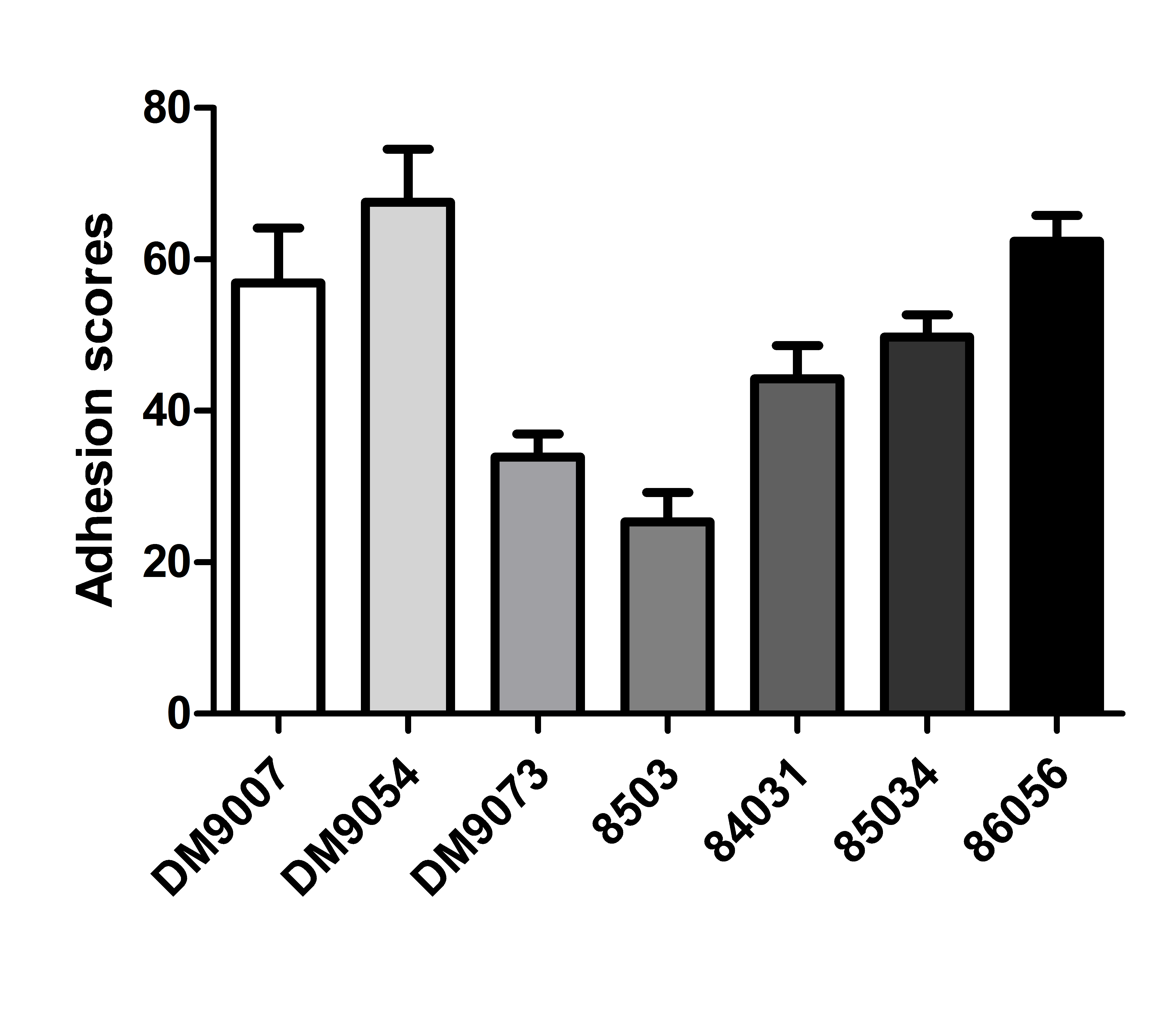

Supplement: S1 Fig — The adhesion scores indicate values of bacteria cells adhered to one Caco-2 cell. All values are means±SD, n = 7. (TIF) [file pone.0138078.s002.tif]
