# Supplementary material for: Co-Administration of Cholesterol-Lowering Probiotics and Anthraquinone from Cassia obtusifolia L. Ameliorate Non-Alcoholic Fatty Liver
Source: PLoS One. 2015 Sep 16;10(9):e0138078. doi: 10.1371/journal.pone.0138078 (PMC4573521; doi:10.1371/journal.pone.0138078)
Supplement: S1 Table — (DOCX) [file pone.0138078.s003.docx]

**Supplementary Table 1. List of primer sequence for reverse transcriptase polymerase chain reaction and the amplicons obtained for different genes**

|  | **Genbank**  **accession** | **Primers**  **(5’–3’)** | **Amplicon**  **size (bp)** |
| --- | --- | --- | --- |
| **CYP7A1** | NM_012942 |  | 381 |
| **Upstream:** |  | GCTATTCTCTGGGCATCTCAAG |  |
| **Downstream:** |  | GAAAGTCAAAGGGTCTGGGT |  |
| **LDL-R** | NM_l75762.2 |  | 154 |
| **Upstream:** |  | GGACACCTGCCAAGATCAAG |  |
| **Downstream:** |  | ATGCTGGAGATGGAGTGGAG |  |
| **HMGCR** | [NM_013134.2](http://www.ncbi.nlm.nih.gov/entrez/viewer.fcgi?db=nucleotide&id=40538851) |  | 151 |
| **Upstream:** |  | GGACCAACCTTCTACCTCAG |  |
| **Downstream:** |  | GCCAATGCTGCCATCAAG |  |
| **FXR** | [NM_021745.1](http://www.ncbi.nlm.nih.gov/entrez/viewer.fcgi?db=nucleotide&id=11120689) |  | 114 |
| **Upstream:** |  | GTGACAAAGAAGCCGCGAAT |  |
| **Downstream:** |  | GCAGGTGAGCGCGTTGTAAT |  |
| **β-acting** | NM_031144 |  | 76 |
| **Upstream:** |  | GGGAAATCGTGCGTGACATT |  |
| **Downstream:** |  | GCGGCAGTGGCCATCTC |  |
