# Supplementary material for: Co-Administration of Cholesterol-Lowering Probiotics and Anthraquinone from Cassia obtusifolia L. Ameliorate Non-Alcoholic Fatty Liver
Source: PLoS One. 2015 Sep 16;10(9):e0138078. doi: 10.1371/journal.pone.0138078 (PMC4573521; doi:10.1371/journal.pone.0138078)
Supplement: S2 Table — aEach value represents mean±SD, n = 7. b- No growth. (DOCX) [file pone.0138078.s004.docx]

**Supplementary Table 2 - The tolerance of candidate strains to biological barriers (Log CFU/mL)**

| **Strains** | **Initial counts^a^** | **pH** | | | **Bile salt (%)** | | |
| --- | --- | --- | --- | --- | --- | --- | --- |
|  |  | **2** | **3** | **4** | **0.1** | **0.2** | **0.3** |
| **DM9007** | 8.31±0.21 | - | 2.49±0.22 | 4.02±0.38 | 8.14±0.31 | 6.67±0.19 | 4.97±0.28 |
| **DM9054** | 9.08±0.12 | 4.09±0.10 | 7.25±0.17 | 9.12±0.24 | 8.89±0.15 | 8.53±0.17 | 8.26±0.23 |
| **DM9073** | 8.75±0.19 | - | 3.07±0.13 | 3.58±0.21 | 8.04±0.33 | 6.89±0.31 | 5.79±0.27 |
| **8503** | 8.82±0.17 | - | 2.40±0.42 | 3.96±0.27 | 2.88±0.23 | 1.72±0.26 | - |
| **84031** | 8.61±0.39 | - | 3.15±0.90 | 6.26±0.82 | 3.14±0.55 | 1.85±0.47 | - |
| **84034** | 9.01±0.19 | 2.17±0.14 | 6.21±0.33 | 7.09±0.48 | 5.02±0.43 | 3.21±0.39 | 1.82±0.28 |
| **86066** | 8.92±0.15 | 3.52±0.12 | 6.95±0.25 | 8.77±0.16 | 7.57±0.28 | 6.39±0.22 | 5.89±0.18 |
| **Strains** | **Initial counts** | **Pepsin (μg/mL)** | | | **Trypsin (U/g)** | | |
|  |  | **0.59** | **0.72** | **1.48** | **33.60** | **52.96** | **72.32** |
| **DM9007** | 8.31±0.21 | 4.31±0.41 | 6.22±0.45 | 8.07±0.57 | 8.53±0.37 | 8.62±0.44 | 8.49±0.51 |
| **DM9054** | 9.08±0.12 | 8.16±0.30 | 8.93±0.22 | 9.02±0.37 | 9.23±0.19 | 9.54±0.25 | 9.37±0.32 |
| **DM9073** | 8.75±0.19 | 8.91±0.32 | 9.08±0.20 | 9.14±0.26 | 9.02±0.57 | 9.11±0.46 | 8.94±0.61 |
| **8503** | 8.82±0.17 | 3.66±0.46 | 6.02±0.38 | 7.91±0.40 | 8.87±0.52 | 8.96±0.67 | 9.04±0.55 |
| **84031** | 8.61±0.09 | 2.97±0.32 | 5.31±0.44 | 7.82±0.41 | 8.78±0.71 | 9.00±0.63 | 9.93±0.61 |
| **84034** | 9.01±0.19 | 6.58±0.50 | 8.40±0.39 | 9.02±0.43 | 8.96±0.37 | 9.14±0.44 | 9.07±0.51 |
| **86066** | 8.92±0.15 | 8.99±0.30 | 9.14±0.28 | 9.15±0.17 | 9.06±0.28 | 9.21±0.33 | 9.05±0.24 |

^a^Each value represents mean±SEM, n=7. ^b^- No growth
