# Supplementary material for: Co-Administration of Cholesterol-Lowering Probiotics and Anthraquinone from Cassia obtusifolia L. Ameliorate Non-Alcoholic Fatty Liver
Source: PLoS One. 2015 Sep 16;10(9):e0138078. doi: 10.1371/journal.pone.0138078 (PMC4573521; doi:10.1371/journal.pone.0138078)
Supplement: S3 Table — Data are mean±SD values (n = 6). Means within a row with different superscript letters are significantly different (P<0.05). (DOCX) [file pone.0138078.s005.docx]

**Effect of *Cassia obtusifolia L.* on fecal properties of SD rats**

| Parameters | Normal control | 0.1 g/kg per day | 0.2 g/kg per day | 0.3 g/kg per day |
| --- | --- | --- | --- | --- |
| Number of fecal pellet | 73.57±3.19^b^ | 73.97±2.59^b^ | 74.91±2.82^b^ | 77.98±3.98^a^ |
| Water content of fecal pellet(ml) | 1.39±0.06^b^ | 1.43±0.03^b^ | 1.45±0.03^b^ | 1.68±0.07^a^ |
| Weight of fecal(g) | 7.08±0.11^c^ | 7.16±0.22^bc^ | 7.28±0.19^b^ | 7.81±0.32^a^ |

Data are mean±SD values (n=6). Means within a row with different superscript letters are significantly different (P<0.05).
